# The generation of a neuronal phase code for space

**DOI:** 10.1002/ctm2.70092

**Published:** 2024-11-13

**Authors:** Eran Stark, Hadas E. Sloin

**Affiliations:** ^1^ Sagol Department of Neurobiology, Faculty of Natural Sciences University of Haifa Haifa Israel; ^2^ Department of Physiology, Anatomy, and Genetics University of Oxford Oxford UK

## 1 RATE AND PHASE CODES FOR SPATIAL REPRESENTATION

Neurons are thought to represent information by spikes, but which specific aspect of spiking activity represents information is debated. Place coding in the mammalian hippocampus provides a useful system for studying forms of neuronal coding. Individual pyramidal cells in the hippocampal CA1 region spike when an exploring rodent is at a particular part of the environment, the “place field” of the cell.^1^ Place cell firing is a “rate code”, where the rate profile is independent of the millisecond timing of individual spikes.

During locomotion, local field potentials in the rodent hippocampus exhibit rhythmic theta (4–11 Hz) oscillations. In many place fields, the spikes follow a specific temporal pattern: when the animal enters the field, spikes occur at the peak of the theta wave, and subsequent spikes occur at progressively earlier phases.^2^ Knowing the phase in which a spike occurs can indicate where the animal is within the place field. This “phase precession” is an example of a “phase code”, in which the phase of the individual spike with respect to the ongoing theta may carry information beyond the firing rate.^3^

**FIGURE 1:**
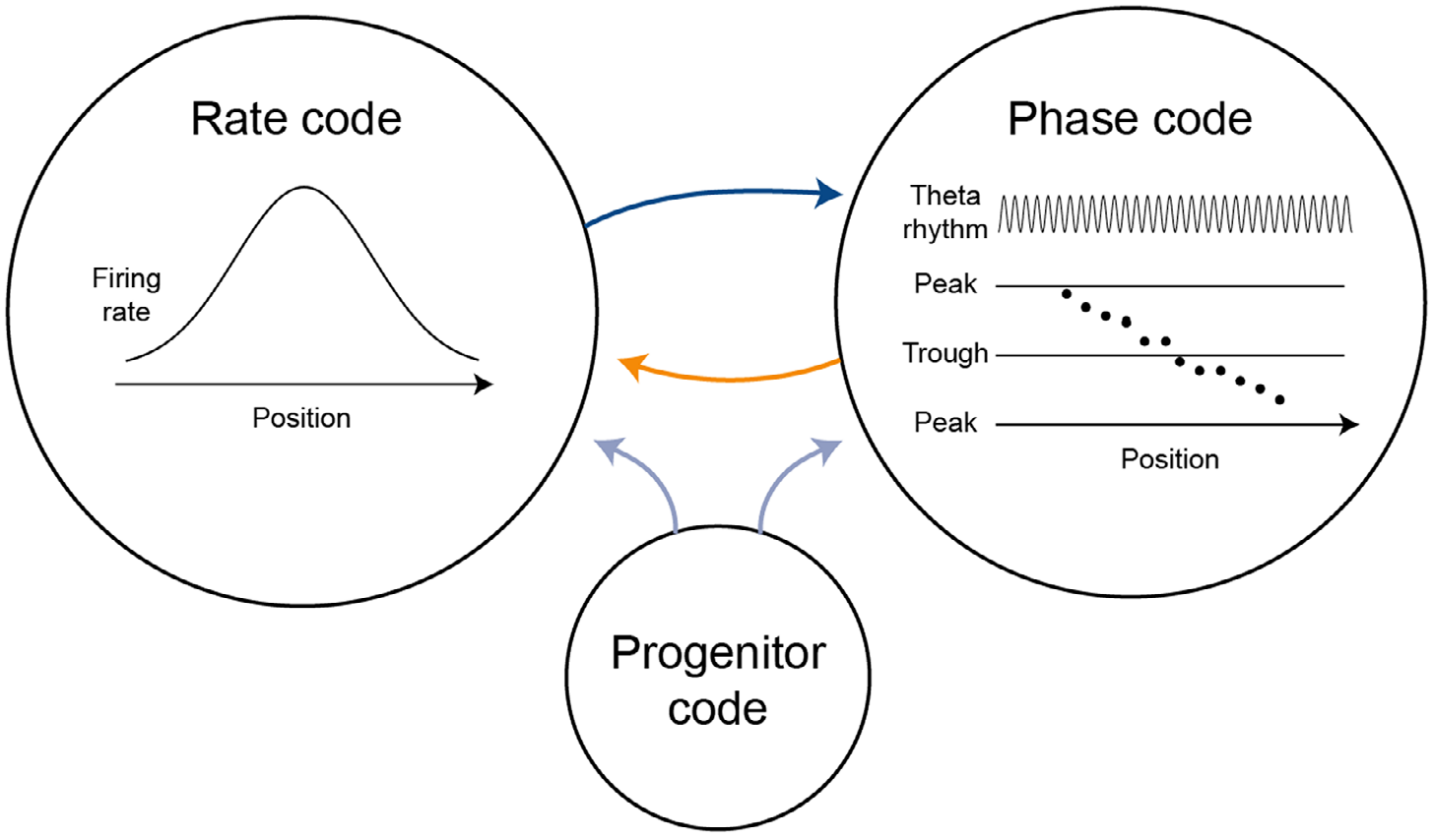
Hippocampal pyramidal cells exhibit increased firing rates within a part of the environment, the “place field”, yielding a rate code for the spatial position. When the animal enters a place field, pyramidal cell spikes occur at the peak of the ongoing theta rhythm and then shift to progressively earlier phases exhibiting “phase precession”, a phase code for the position. Whether and which code is converted into another has been the topic of numerous studies in the last three decades.

The observation that both rate and phase codes may carry information about the same parameter in the same neuron provides an opportunity for understanding how these codes are generated. A priori, there are at least three options (Figure 1): a rate code might be somehow converted into a phase code^4^; a phase code may be converted into a rate code^5^; or a third, “progenitor” code could generate both.^6^ Work in the past three decades provided evidence for all three possibilities and for various combinations thereof, and numerous models were proposed for generating phase precession.^7^

## 2 A CLOSED‐LOOP APPROACH FOR DETERMINING THE SOURCE OF A PHASE CODE

We combined electrophysiological recordings with optogenetic manipulations in freely‐moving mice to approach the problem of the generation of rate and phase codes in CA1 pyramidal cells. We reasoned that imposing one code on the system may determine whether the codes are interdependent, and possibly constrain the generative mechanisms. Because multiple prior studies suggested that the phase precession observed in CA1 is inherited from other regions,^7^ we chose to impose a rate code on individual pyramidal cells in CA1.

The induction of an artificial rate code presents several technical challenges. First, we developed hardware to manipulate the activity of individual neurons deep in the brain of freely‐moving subjects.^8^ We invented multi‐site/multi‐colour optoelectronic devices called “diode‐probes” in which light from multiple miniature sources is emitted near the recording electrodes. Second, we coupled the activation of CA1 pyramidal cell spiking with the actual kinematics of the mouse. We developed a system called “Spotter” that tracks subject movement in real time,^9^ and used the system to implement a mathematical model of a 2D place field modulated by head orientation.

## 3 CONVERSION OF AN IMPOSED RATE CODE TO A PHASE CODE

**FIGURE 2:**
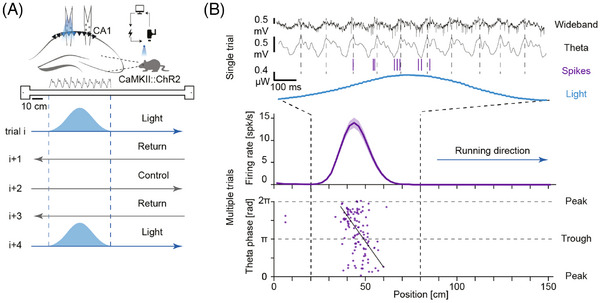
Focal activation of pyramidal cells in hippocampal region CA1 generates an artificial rate code which is spontaneously converted into a synthetic phase code. (A) Experimental design. A high‐density diode‐probe^8^ is implanted in CA1 of a freely moving mouse that expresses ChR2 in CA1 pyramidal cells. During alternate same‐direction runs on a linear track, a closed‐loop system^9^ monitors the position and head orientation of the subject and drives illumination on one or more shanks of the array. (B) The firing rate of an individual pyramidal cell follows the light pattern. Although the system generates the light pattern irrespective of the ongoing theta oscillations, the individual spikes occur at progressively earlier theta phases.^10^ **Top**, a single pass through the artificial place field. **Middle**, rate profile of the artificial place field, averaged over multiple light trials. **Bottom**, phase of every spike at every position. *Source*: Adapted.^10^

We trained mice to run back and forth on a linear track. In every experimental session, we selected a random location along the track for imposing a rate code. On every other lap in a given direction (e.g., left‐to‐right run), we closed the loop on mouse kinematics by turning on the optogenetic feedback system^8^, ^9^, ^10^ (Figure 2A). The result was the induction of a rate code in CA1 pyramidal neurons, yielding artificial place fields (Figure 2B, middle). Artificial fields appeared in all tested mice (286/1095 pyramidal cells, 26%), meeting the technical challenge of creating place fields based on an arbitrary model and actual animal kinematics.

The key finding was that in many cases (105/286 artificial fields, 37%), the imposed rate code also generated a phase code.^10^ Specifically, spikes of pyramidal cells that exhibited an artificial place field did not occur at random phases of the ongoing theta oscillations (Figure 2B). Instead, artificial place field spikes exhibited synthetic phase precession: spikes induced at the beginning of the artificial field occurred around the peak of theta; spikes induced at the middle of the field occurred towards the theta trough; and spikes near the end of the field again occurred close to the theta peak.^10^ Thus, spikes generated by inducing place field‐like activation exhibited phase precession, indicating that a rate code imposed on CA1 pyramidal cells is spontaneously converted into a phase code.

## 4 IMPLICATIONS AND OPEN QUESTIONS

In some cases, the closed‐loop optogenetic place fields overlapped preexisting place fields that exhibited spontaneous phase precession. In those cases, the imposed drive interfered with and slowed down the precession, indicating that precession is not inherited from an upstream region.^10^ Thus, the results show that phase precession can be generated locally in CA1, as opposed to being inherited from other sources or regions. Repeating the experiment in the posterior parietal cortex induced artificial fields but no synthetic precession.^10^ However, it is unclear whether the phase precession observed in other parts of the hippocampal formation is also generated locally within each structure.

By comparing the predictions of multiple generative models with the experimental observations, we narrowed down possibilities to a single class of models. In dual oscillator models,^2^, ^7^ a slower oscillator (the theta rhythm) co‐occurs with a faster oscillator triggered within the place field. We designed simple dual oscillator models in which the pyramidal neuron itself is the faster oscillator.^10^ In our models, the faster intracellular rhythm is generated by an interaction between a persistent sodium current and another current: either a slowly‐activated potassium current or a slowly‐inactivated sodium current. While only the dual oscillator models recapitulated the experimental observations, other generative mechanisms may be identified in the future.

Can a phase code generate a rate code? It remains to be explored whether imposing a phase code on the system spontaneously generates a rate code. The discussed work^10^ and the future assessment of phase‐to‐rate code conversion may uncover additional mechanisms of information representation by different coding schemes (Figure 1). However, whether rate or phase codes are directly used by the brain to drive behaviour remains unknown, and will require dedicated investigation.

## CONFLICT OF INTEREST STATEMENT

The authors declare no conflict of interest.
